# Protective and Modification Strategies for Instrument Wood: A Critical Review

**DOI:** 10.3390/polym18060758

**Published:** 2026-03-20

**Authors:** Qingdong Liang, Junfei Ou

**Affiliations:** 1 School of Education, Jiangsu University of Technology, Changzhou 213001, China; liangqd6100@gmail.com; 2 School of Materials Engineering, Jiangsu University of Technology, Changzhou 213001, China

**Keywords:** wood modification, protective coatings, acoustic performance, sustainability, performance trade-offs

## Abstract

Wood is the quintessential material for musical instruments due to its superior acoustic properties. However, its inherent susceptibility to environmental degradation—including moisture-induced dimensional changes, photodegradation, and biological attack—presents a fundamental challenge that treatment strategies must address. This critical review systematically examines recent advances in wood modification and surface protection technologies for musical instruments, encompassing chemical and thermal modification, protective coatings, physical densification, and biological treatments. Drawing on studies published over the past two decades, this review synthesizes current knowledge on how these interventions affect wood’s acoustic performance, dimensional stability, mechanical integrity, and long-term durability. A central finding is that treatment outcomes are highly species-specific and involve complex performance trade-offs: acoustic optimization often comes at the expense of mechanical strength or dimensional stability, and the optimal solution varies depending on the functional requirements of specific instrument components (e.g., soundboards versus fingerboards). Emerging bio-based and nanocomposite coatings show promise for enhancing environmental resistance, but their acoustic implications remain largely unexplored. Furthermore, most research remains at the laboratory scale, with limited validation on full instruments and a notable absence of long-term performance data under natural aging conditions. To advance the field from empirical trial-and-error toward predictive, knowledge-based design, this review identifies three priority areas for future research: (1) establishing cross-scale “treatment-structure-performance” correlation models that bridge molecular-level modifications to instrument-level acoustic outcomes; (2) developing intelligently engineered surface systems capable of multi-objective synergistic optimization; and (3) creating comprehensive assessment standards that encompass acoustics, durability, and sustainability. By systematically synthesizing current knowledge and identifying critical gaps, this review provides a foundation for more targeted, interdisciplinary research in instrument wood protection.

## 1. Introduction

Wood, a natural biomass material refined over thousands of years, remains the primary material for string, wind, and many folk instruments. Its distinctive cellular structure, anisotropic nature, and complex composition of cellulose, hemicellulose, and lignin confer exceptional vibrational and acoustic qualities [1]. From the legendary tones of Stradivarius violins to the resonant brightness of modern concert pianos, the acoustic properties of wood define an instrument’s character. However, this exceptional acoustic medium is inherently fragile and susceptible to environmental fluctuations: as a hygroscopic porous material, it readily undergoes dimensional changes in response to humidity variations, leading to cracking or warping; its organic components are subject to photodegradation under UV light, resulting in discoloration and surface damage; and it faces continuous threats from biological organisms such as fungi and insects [1]. Consequently, surface protection for instrument wood is not merely an aesthetic consideration but a critical technical intervention essential for maintaining structural integrity, acoustic stability, and longevity.

The methods employed to achieve this protection have evolved significantly over time. Traditionally, instrument finishing relied on natural resins, oils, and waxes, based largely on empirical knowledge passed down through generations of artisans [2,3]. Historical analyses of Cremonese school violins, including those by Stradivari, have revealed that these classic instruments were finished with complex oil-resin varnish systems, typically comprising mixtures of linseed or walnut oil with natural resins—knowledge derived from chemical characterization studies of historical instrument coatings [4]. These formulations evolved through centuries of craft tradition, with specific recipes often closely guarded by individual workshops. In the 20th century, synthetic coatings such as nitrocellulose and polyurethane gained popularity due to their rapid drying time, glossy finish, and ease of application [2]. However, these traditional and modern coatings have frequently been regarded as passive layers applied to the instrument, with their complex effects on the underlying wood’s vibrational behavior long underestimated—a gap that recent studies on coating aging and acoustic performance have begun to address [5].

This growing recognition of coatings’ active role coincides with broader technological developments. Over the past two decades, the integration of materials science, interface engineering, and acoustic measurement techniques has catalyzed a paradigm shift in instrument wood protection—from passive “covering protection” to active “surface engineering”—aiming to modulate or enhance acoustic properties while providing excellent durability through specific chemical modification, physical coating, or structural design [6,7].

Regardless of the approach chosen, any intervention inevitably alters the wood’s physical characteristics. Any treatment applied to the wood surface—whether a chemical substance that penetrates and alters bulk properties or a coating that adheres to the surface—modifies critical physical parameters including density, elastic modulus, and internal friction [7,8]. A classic challenge is that increasing coating thickness for enhanced water resistance may add excessive mass and dampen high-frequency vibrations; incorporating hard nanofillers to improve abrasion resistance may increase overall stiffness and alter the instrument’s vibrational modes [9]. Further complicating the issue, different wood species (e.g., spruce versus maple), and even different sections of the same piece of wood (heartwood versus sapwood), may respond uniquely to identical treatments [10,11].

These complexities are compounded by the fragmented nature of the research community itself. The research landscape in this field remains fragmented. On one hand, acoustics researchers focus on how modifications affect vibration spectra; on the other, materials scientists strive to develop superior protective coatings. Sufficient interaction and dialog between these communities is lacking [7,12]. Additionally, the absence of standardized evaluation protocols, a scarcity of long-term performance data, and increasing pressure for environmental sustainability collectively hinder progress in this field [12].

These interconnected challenges—the fragmentation of research efforts, the absence of standardized evaluation protocols, the lack of long-term performance data, and the growing imperative for environmental sustainability—represent critical gaps in the current literature. Addressing these gaps requires a comprehensive synthesis that integrates findings across disciplines and critically evaluates the state of knowledge on how surface treatments affect both protection and acoustic function.

This review provides a critical examination of protective and modification strategies for instrument wood, with particular attention paid throughout to the trade-offs between acoustic performance, long-term durability, and environmental sustainability across different technological approaches. To this end, the article surveys the technological landscape through a deliberately structured progression of five subsequent sections. Section 2 examines chemical and thermal modifications—techniques that penetrate the wood bulk to alter its fundamental properties from within. Section 3 turns to protective coatings which interface with the wood externally, adding a discrete layer that participates actively in the vibrational system. Section 4 introduces physical and biological treatments, which operate through yet another mechanism: directly reconfiguring the wood’s structure through densification, fungal action, or exploitation of natural variability. Section 5 then examines the emerging paradigm of multifunctional integration, wherein multiple protective and acoustic objectives are addressed simultaneously within unified treatment systems. Finally, Section 6 synthesizes the gaps and challenges identified across all preceding sections—the scale mismatch between laboratory specimens and complete instruments, the absence of long-term performance data, the fragmentation of research efforts, and the unresolved conflict between multifunctionality and acoustic transparency—into prioritized research directions aimed at advancing the field from empirical craft toward predictive science.

## 2. Chemical and Thermal Modification

Among the various approaches to enhancing wood performance, chemical and thermal modifications represent some of the most established techniques. Chemical and thermal modifications have long been conventional engineering techniques for improving the dimensional stability and biological durability of wood. The underlying principle involves altering the structure and properties of wood cell wall components (particularly hemicellulose and lignin) through heat or chemical reagents, reducing the number of hygroscopic hydroxyl groups, or creating stable cross-linked networks. However, when applied to instrument wood—which exhibits exceptional sensitivity to vibrational behavior—the outcomes are considerably more complex than simple improvements or deteriorations, exhibiting pronounced dependence on wood species and treatment parameters.

### 2.1. Species Specificity and Performance Trade-Offs in Thermal Modification

The species-dependent nature of treatment outcomes is particularly evident in thermal modification studies. Studies on thermal modification provide substantial evidence for species-specific responses. Aydogmus et al. [10] demonstrated considerable variation across species: when spruce, maple, and mahogany were heated to 210 °C for 90 min, acoustic properties such as sound velocity and acoustic radiation changed differently—decreasing for spruce, improving for maple, and remaining relatively unchanged for mahogany. This suggests that thermal modification cannot be considered a universally reliable method for enhancing acoustic quality. Notably, despite these divergent acoustic responses, all three species exhibited reduced bending strength. This inverse relationship between acoustic improvement and mechanical integrity has important implications for string instrument components such as soundboards and necks. Similar trade-offs between acoustics and mechanics were observed in Paulownia wood used for Ruan instruments, where certain modifications increased the modulus of elasticity but typically at the cost of increased brittleness [13].

Beyond species specificity, treatment parameters play a critical role in determining outcomes. The effects of thermal modification are highly dependent on temperature, duration, and post-treatment conditions. Merhar et al. [14] investigated the dynamic properties of thermally treated spruce under various temperatures (180–230 °C) and humidity conditions (20–88% RH). They found that high-temperature modification (230 °C) resulted in an average density reduction of 16%, accompanied by decreases of approximately 9.8% and 9.7% in the dynamic modulus of elasticity and shear modulus, respectively. However, sound velocity and sound radiation coefficient increased with rising environmental humidity (by 1–3% and 10–15%, respectively). This apparent paradox—reduced modulus yet enhanced sound radiation under moist conditions—suggests that thermal treatment induces a viscoelastic shift in the cell wall, reducing damping and allowing more free vibration at specific frequencies. The intensity of modification emerges as a crucial variable. Buchelt et al. [15] reported that for spruce, mild thermal modification (180 °C) does not produce significant changes in vibrational characteristics across a wide range of humidity conditions, with variations in tan δ primarily attributable to humidity rather than thermal modification. This underscores the importance of “modification intensity”: insufficient treatment yields no discernible acoustic effect, while excessive treatment may compromise structural integrity. Optimal treatment windows depend on the wood species and intended application.

### 2.2. Potential and Uncertainty in Chemical Modification

**Figure 1:**
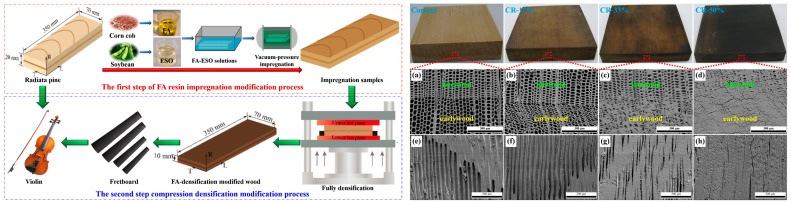
Schematic of the preparation procedure of the furfuryl alcohol-densified wood (**left**). Scanning electron microscope images of transverse and radial sections of the control wood (**a**,**e**) and modified wood with compression ratios (CRs) of 17% (**b**,**f**), 33% (**c**,**g**), and 50% (**d**,**h**) (**right**) [17].

While thermal modification operates through relatively non-specific pyrolytic processes, chemical modification offers a more targeted approach. In contrast to the “pyrolytic reconstruction” of thermal modification, chemical modification offers a more precise molecular engineering approach through reactions (such as esterification and etherification) between reagents and wood cell wall components. Aydogmus et al. [16] investigated the effects of propionic anhydride treatment on the acoustic properties of five different instrument woods. Results indicated that dimensional stability improved substantially in all woods as weight percent gain (WPG) increased with longer reaction time. However, no consistent trends emerged regarding sound velocity, vibrational frequencies, or stiffness; these parameters varied according to wood species and treatment duration. This variability likely stems from differences in wood anatomy and chemistry. This uncertainty likely stems from interspecific differences in cellular architecture and chemical composition, which influence reagent penetration and distribution within the wood structure. Conversely, composite modification involving furfuryl alcohol (FA) resin impregnation followed by compression of Radiata pine demonstrated pronounced beneficial effects, significantly increasing dynamic modulus to meet fretboard requirements [17] (Figure 1). This highlights the distinction in design logic and outcome predictability between purely chemical and combined physical–chemical approaches.

Among the diverse chemical reagents explored, boron-based compounds have attracted particular attention. Among various chemical modification strategies, boron-based treatments have attracted attention for their environmental compatibility [18]. Boric acid and borates are established as effective wood preservatives, yet their acoustic effects remain incompletely characterized. Research indicates that treatment of spruce with 1% disodium octaborate tetrahydrate/boric acid increases hygroscopicity and reduces dimensional stability; unexpectedly, the internal friction coefficient (tan δ) decreases, improving the conversion efficiency of sound waves to mechanical energy. Fourier transform infrared spectroscopy combined with principal component analysis revealed complex interactions between boron compounds and wood constituents. These findings point toward the possibility of truly multifunctional modifiers. This suggests the possibility of developing “multifunctional modifiers” that simultaneously provide protection and acoustic enhancement through targeted, non-disruptive interactions with the cell wall. Another significant approach is acetylation, which effectively improves dimensional stability and decay resistance, but its acoustic effects remain contested. Some studies report reduced elasticity, while others suggest that acetylated wood maintains favorable acoustic properties even under humid conditions [19]. These discrepancies may reflect variations in treatment intensity and post-treatment conditioning, emphasizing the importance of temporal factors in chemical modification processes.

### 2.3. Current Limitations and Gaps in Mechanistic Understanding

Despite the insights gained from these studies, significant limitations persist. Although studies on chemical and thermal modification have demonstrated species-specificity and performance trade-offs, the accumulated knowledge falls considerably short of providing predictable, adjustable, and durable “tuning” methods for instrument wood. The primary limitation is the scale of research: most experiments conduct vibrational tests on small, uniformly sized specimens [10,14,15,18], which differ substantially from actual instrument components such as arched, variably thick soundboards with rib supports, or necks subjected to complex loading. Whether improved performance in laboratory specimens translates to enhanced sound quality in complete instruments remains an unverified hypothesis. Component-level or instrument-level validations are rare; one exception is the evaluation of European wood species for classical guitar neck stability under sustained string tension [20].

A second, equally critical limitation concerns the absence of long-term performance data. How the chemical stability, potential interface degradation, and acoustic properties of modified wood will evolve over years or decades of humidity cycling, temperature fluctuations, and light exposure is largely unknown. Will the new chemical bonds or network structures render wood more susceptible to embrittlement or fatigue under prolonged stress? No natural aging data exist to substantiate claims regarding the long-term efficacy of modification treatments. Accelerated aging tests typically fail to faithfully reproduce the complex, gradual, and synergistic effects of real-world environmental exposure [1].

Perhaps most fundamentally, mechanistic understanding at the cell wall and nanoscale remains rudimentary. Current research largely confines itself to establishing empirical correlations between macroscopic performance parameters (density, modulus, damping) and treatment conditions (temperature, time, concentration). Explanations of underlying mechanisms—such as how heat or chemicals alter cellulose microfibril arrangement, modify the rheology of the hemicellulose-lignin matrix, or affect interfacial bonding between cell wall components—are comparatively scarce. This lack of mechanistic insight impedes the transition from empirical trial-and-error to rational design. Bridging this gap requires enhanced collaboration among wood science, chemistry, and computational materials science.

To provide a visual synthesis of the complex trade-offs and species- or parameter-dependent results discussed above, Figure 1 presents a comparative assessment of five major modification technologies. The radar chart evaluates each technology across five dimensions: dimensional stability, acoustic impact potential, mechanical strength retention, ecological sustainability, and process control. It graphically illustrates that no single technology excels in all dimensions. For example, furfuryl alcohol resin impregnation combined with compression exhibits a strong, balanced profile, whereas boron compound treatment demonstrates pronounced trade-offs, prioritizing acoustic potential and sustainability over dimensional stability. This visualization underscores that technology selection inherently involves prioritizing specific performance attributes based on application requirements.

**Figure 2:**
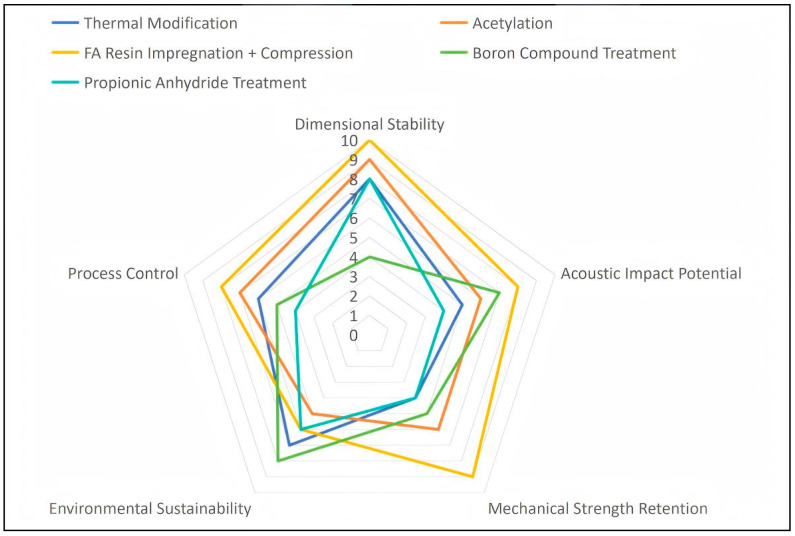
Authors’ own elaboration based on the reviewed literature. Performance trade-offs among major wood modification technologies for musical instruments. Radar chart assesses five dimensions (1–10 scale, with higher scores indicating superior performance). Note: the scoring of “acoustic impact potential” and “mechanical strength retention” represents general trends and should be interpreted with consideration of component-specific requirements. For soundboards where acoustic responsiveness is paramount, high acoustic potential may be prioritized over mechanical strength; for structural components such as fingerboards and necks, mechanical strength retention becomes critical. This visualization illustrates relative trade-offs rather than prescribing universal optima. No single technology excels across all dimensions. Data for thermal modification were synthesized from references [10,14,15]; FA resin impregnation with compression from [17]; propionic anhydride treatment from [16]; acetylation from [19]; and boron compound treatment from [18].

The relevance of the performance trade-offs shown in Figure 2 becomes evident when considering specific instrument components. A fingerboard, as studied by Liu et al. [17], requires both high mechanical strength to withstand string tension and optimized acoustic properties for sound transmission—demands that can be simultaneously addressed through furfuryl alcohol resin impregnation combined with densification, which enhances dynamic modulus, shear modulus, and acoustic radiation efficiency. A soundboard, by contrast, places paramount importance on acoustic radiation, where acoustic impact potential may outweigh mechanical strength considerations. This component-specific perspective reveals that the optimal technology is not universal but depends on the functional requirements of the intended application.

While chemical and thermal modifications alter wood properties from within, an alternative and equally important approach involves applying protective layers directly to the surface. The following section examines coating technologies which interface with the wood externally yet play an equally critical role in determining acoustic outcomes.

## 3. Protective Coatings

As established in the preceding section, modifications that penetrate the wood bulk can substantially alter acoustic behavior. However, the most direct and historically prevalent form of intervention operates at the surface itself. Coatings constitute the most direct protective layer on instrument wood surfaces. The principal challenge lies in creating an effective barrier against moisture, UV radiation, stains, and physical damage without unduly restricting the wood’s vibrational freedom. This delicate balancing act has characterized the evolution of coating technologies from traditional varnishes to advanced functional coatings.

### 3.1. Traditional Varnishes: Complex Interactions Within Empirical Traditions

Understanding the behavior of traditional varnishes provides essential context for evaluating newer technologies. Traditional varnishes—including nitrocellulose, oil-based resins, and alkyd resins—have long been employed in instrument making, yet their effects have often remained shrouded in empirical lore rather than quantitative understanding. Stanciu et al. [3] conducted a systematic investigation that elucidated these effects. Nitrocellulose, oil-based, and spirit varnishes were applied to Norway spruce resonance plates in layers ranging from 5 to 15. Results revealed dramatic reductions in wood lightness: approximately 17% after one coat, 50% after five coats, and nearly 70% after fifteen coats. Critically, strong correlations emerged between changes in color parameters and the anatomical characteristics of the wood (annual ring width, earlywood/latewood ratio)—most pronounced for oil-based varnishes and weakest for nitrocellulose. This indicates that different resins interact with the wood surface through distinct wetting, penetration, and chemical reaction mechanisms, producing varied interface structures and optical effects. From a performance perspective, oil-based alkyd resins tend to impart greater flexibility and fullness to the finish but require extended drying times, whereas nitrocellulose lacquers dry rapidly and form hard films that may exhibit brittleness [21]. Such fundamental material property differences inevitably translate into distinct effects on sound energy transmission.

Acoustically, coatings are far from passive additions to the instrument. Acoustically, coatings introduce additional mass, stiffness, and damping. Their effects are not invariably detrimental: an exceptionally thin, hard coating may slightly enhance high-frequency response by functioning as a stiff shell; conversely, a thick, soft coating may excessively dampen vibrations through substantial energy dissipation and potential restriction of panel flexural deformation. The critical insight is that the coating no longer functions as a passive, inert covering but becomes an active participant in the instrument’s vibrational system [2,5]. Its thickness, elastic modulus, damping characteristics, and adhesion to the wood substrate collectively influence how it modifies the system’s overall vibrational modes and energy radiation efficiency. Historical instruments provide compelling evidence for this active role. Studies of historical instruments support this conceptualization. Analysis of Cremonese school violin coatings has revealed that the ancient oil–resin varnish systems are not only compositionally complex but also exhibit specific interactions with the wood substrate that may contribute to their legendary sound quality [4]. This suggests that modern coating research should not focus exclusively on novel materials but should also seek to extract design principles from historical knowledge regarding interface engineering.

### 3.2. Bio-Based and Nano-Enhanced Coatings: Frontiers of Sustainability and Functional Integration

**Figure 3:**
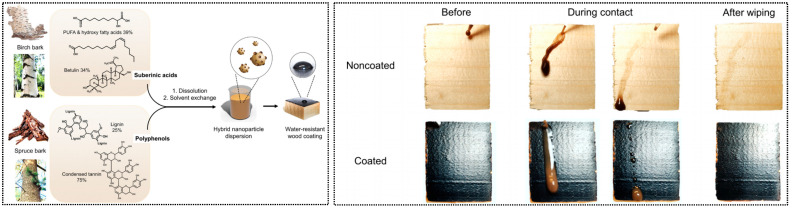
Production of bark-derived water-resistant wood coating. Birch outer bark and spruce bark were used to extract suberinic acids and polyphenols, which were then combined to form an aqueous dispersion coating (**left**). Digital photograph of uncoated and coated (hybrid NPs with 40% polyphenols) spruce wood before and after contact with muddy water (**right**) [22].

Contemporary coating research has expanded beyond traditional formulations to address sustainability concerns. Concerns regarding VOC emissions from solvent-based coatings and dependence on petroleum resources have driven the development of high-performance, water-based, and bio-based coatings. Wang et al. [22] developed a fully bio-based waterproof coating derived entirely from bark: suberinic acids and polyphenols extracted from birch and spruce bark form an aqueous suspension that, upon thermal curing on wood, exhibits superior water resistance compared to commercial alkyd emulsions (Figure 3). Inspired by the natural barrier function of bark, this work achieved a closed-loop utilization of wood processing by-products for wood protection. Similarly, Grigorescu et al. [23] nanosized lignin—a paper industry by-product—combined it with ZnO nanoparticles, and embedded the mixture in a biodegradable PHB matrix to create a multifunctional composite coating. This coating substantially enhanced wood hydrophobicity (contact angle reaching 145°), while demonstrating effective UV protection and antifungal properties. These studies exemplify advanced approaches to sustainable protection through biomimetic design and valorization of waste materials. Along similar lines, a phytic acid–silica flame retardant system incorporating lignin and silica demonstrates the potential for converting biomass into high-performance fire-resistant coatings [24]. Such bio-based solutions address the urgent demand for environmentally friendly materials in the instrument industry [12].

Parallel to these developments, nanomaterials offer unprecedented control over coating properties. Concurrently, nanomaterials provide powerful tools for fine-tuning coating properties. Cellulose nanofibrils (CNF) are particularly valued for their exceptional specific strength, biodegradability, and compatibility with wood. Zigon et al. [25] incorporated CNF into an aqueous PVA solution for wood coating applications. CNF addition increased the indentation hardness of pure PVA films, and the coating penetrated 100–200 μm into the wood, becoming well-anchored within cells while imparting UV-blocking properties. Nano-SiO_2_ modified with the silane coupling agent KH-550 has been shown to enhance the hardness, abrasion resistance, and hydrophobicity of water-based wood coatings [9]. However, a critical question remains conspicuously under addressed. However, an inevitable question arises: what effects do these nanofillers or bio-based resins—designed to improve protective performance—have on the acoustic properties of instrument wood? Have these effects been systematically investigated? Do the denser, stiffer films they create introduce undesirable damping? Do they facilitate or impede vibrational energy transmission through the wood? These questions remain largely unaddressed in current coating development, creating a disconnect between protective performance research and acoustic impact assessment. A rare exception is the work by Tamantini et al. [26], who incorporated cellulose nanocrystals (CNC) into a waterborne acrylic coating for instrument wood and evaluated its decay resistance. Although acoustic testing was not performed, the study’s paradigm—considering coating influence on overall instrument performance during the design process—represents a step in the right direction.

### 3.3. Coating Aging and the Uncharted Territory of Performance Evolution

Beyond initial performance, the long-term behavior of coatings presents equally important challenges. Coating aging behavior constitutes a major concern for long-term instrument preservation yet remains poorly understood. Upon exposure to UV radiation, hygrothermal cycling, oxygen, and pollutants, coatings undergo photo-oxidative degradation, hydrolysis, micro-cracking, chalking, and potentially delamination from the substrate [5]. This progressive degradation compromises protective function while continuously altering acoustic properties as the coating evolves in mechanical characteristics and may partially detach from the instrument [5]. Wan et al. [5] employed machine learning approaches to predict the effects of varnish aging on acoustic damping and structural stability of stringed instruments, highlighting the extreme complexity of this problem. The methods used to study aging introduce their own complexities. However, most existing coating durability studies rely on accelerated aging tests (e.g., QUV chambers). While these accelerated conditions are presumed to simulate real-world aging processes experienced by instruments during playing and conservation (humidity fluctuations, light exposure, temperature variations), their fidelity to natural aging has not been systematically verified [1,27]. The absence of reliable correlation models between “accelerated aging” and “natural aging” renders lifetime predictions based on short-term accelerated tests highly uncertain, limiting their utility for guiding assessment and intervention decisions regarding original coatings on historical instruments during conservation practice. Some emerging coating systems incorporate self-healing capabilities, such as those containing microencapsulated wood oil [28]. While this approach offers potential for mitigating aging damage, its efficiency and effects on aged acoustic properties require further investigation.

It must be acknowledged, however, that the preference for natural aging data must be balanced against practical realities. Natural aging studies spanning decades are inherently incompatible with contemporary research and product development timelines. Accelerated aging tests, despite their imperfect correlation with natural aging, remain indispensable for generating timely performance predictions and comparative data. The challenge, therefore, is not to abandon accelerated testing but to develop more robust correlation models that bridge the gap between laboratory-accelerated conditions and real-world aging processes—a goal requiring systematic, long-term monitoring of naturally aged specimens to serve as calibration references.

Table 1 gives an overview of the new coating systems and shows a trend towards sustainability by using bio-based materials, by-products, and water-based formulations. However, the column about acoustic impact shows a big and steady gap in what people know. These coatings are made to have great protective qualities, such as being good at keeping water out, blocking harmful sun rays, and becoming harder, but it is still mostly unknown how they might affect how instruments sound when played. Further, this gap is not just a matter of not having enough information, it is a basic mismatch in what we care about: the science of making things last and stay dry and eco-friendly has not been mixed with the part about making music sound good. Those same qualities that make them good candidates for sustainable coatings—tightly woven bio-film networks, or super hard and scratch proof nanofiller layers—might also cause some unwanted dampening or stiffness. Therefore, the table indicates that the current “green revolution” in coating technology comes with a notable warning for luthiers; it will only be embraced if there is future research that purposefully closes this material–acoustic gap.

Coatings represent the most direct form of surface intervention, applying a discrete layer atop the wood substrate. However, a fundamentally different class of approaches modifies acoustic behavior, not through added layers, but through physical restructuring or biological alteration of the wood itself, as discussed in the next section.

## 4. Physical and Biological Treatments

While coatings modify the wood surface through applied layers, physical and biological treatments operate through distinct mechanisms that directly reconfigure the wood’s structure. Beyond conventional chemical modification and coating techniques, certain physical and biological treatment approaches offer distinct mechanisms for modulating the acoustic characteristics of instrument wood. These methods typically act directly on wood density, porosity, or cell wall structure.

### 4.1. Compression Densification and Resin Impregnation for Property Enhancement

The need for sustainable alternatives to traditional hardwoods has driven interest in densification technologies. Certain instrument components—such as fretboards and necks—require high density, hardness, and dimensional stability, traditionally achieved through the use of dense tropical hardwoods. In the pursuit of sustainable alternatives, modification of fast-growing plantation wood has emerged as a research priority. Liu et al. [17] conducted a comprehensive investigation on radiata pine, initially saturating specimens with furfuryl alcohol (FA) resin followed by radial compression at ratios of 17%, 33%, and 50%. Results demonstrated that wood treated with FA and compressed at 50% compression ratio exhibited a dynamic modulus of elasticity (D′) of 25.44 GPa and dynamic shear modulus (G′) of 4.08 GPa, both exceeding the minimum requirements for conventional fretboard wood (D′ ≥ 16.22 GPa, G′ ≥ 2.23 GPa). Concurrent improvements were observed in sound velocity, sound radiation coefficient, and acoustic conversion efficiency, indicating significant potential for replacing traditional expensive hardwoods in fretboard production. The underlying mechanism involves structural collapse combined with resin reinforcement. This approach fundamentally involves the partial collapse of the porous structure of low-density wood combined with resin reinforcement, achieving substantial enhancement of physical and mechanical properties. The method is not restricted to softwoods; modification of species such as Ailanthus, with potential for instrument backs and sides, requires understanding of natural radial and longitudinal property variations to select optimal wood sections for treatment [11]. The search for sustainable alternative species for specific components such as soundboards and fretboards constitutes an active research domain that informs candidate selection for these intensive modification processes [31].

### 4.2. Fungal Biotreatment: Nature’s “Acoustic Engineer”

While densification represents a top-down engineering approach, biological methods offer a bottom-up alternative. While wood-decaying fungi are typically regarded as destructive, certain white-rot fungi can function as “biological engineers.” The work of Kim et al. [32] is particularly noteworthy: they exposed alder and soft maple to eight different white-rot fungal species, including Trametes versicolor. After only four weeks, certain fungal species substantially altered the acoustic properties of the wood. For example, Trametes versicolor and Ceriporia lacerata increased the acoustic radiation damping coefficient (A) of alder wood by 33.0% and 21.0%, respectively, and acoustic conversion efficiency (ACE) by 50.4% and 37.6%, respectively. Similar, though slightly less pronounced, improvements were observed in soft maple. The proposed mechanism involves selective degradation of cell wall components. The proposed mechanism involves selective degradation of hemicellulose and lignin components by the fungi, subtly modifying the wood’s density-acoustic relationship and enhancing its sound radiation capacity. This biotreatment approach offers a gentler, more environmentally friendly, and potentially cost-effective method for modifying wood acoustics, although challenges remain regarding process control, uniformity, and long-term stability. Historical practices provide precedents for biological approaches. It is noteworthy that certain cultural traditions have historically employed fermentation processes to enhance the acoustic qualities of woods such as walnut or pine, likely involving microbial activity though with less clearly defined mechanisms than controlled fungal inoculation [33,34]. These traditional practices point toward long-standing, albeit empirical, recognition of biological assistance in wood “seasoning” for instrument making.

### 4.3. Influence of Internal Structural Variations and Processing

Underlying all treatment approaches is the inherent variability of wood itself. Instrument makers have long recognized that natural variability within wood of the same species—including ring width, grain orientation, and proportion of juvenile wood—significantly influences final instrument sound [35,36]. Modern research has begun to quantify these effects. Hassan et al. [11] investigated the variation in the acoustical–physical properties of Ailanthus altissima wood along longitudinal and radial directions of the trunk, finding that density and dynamic modulus initially increased then decreased from bottom to middle, while acoustic conversion efficiency exhibited the opposite trend. This indicates that even within a single tree, wood from different positions is suited to different acoustic functions. Guiman et al. [37] specifically examined the influence of grain orientation on wood sound absorption characteristics, discovering substantial differences in sound absorption coefficient, reflection, and impedance ratio among spruce and maple across the three principal sections (transverse, radial, tangential). These studies underscore that the success of any surface treatment technology is contingent upon the wood’s inherent, highly variable structural foundation. Consequently, future treatment approaches may need to integrate wood selection and processing more intimately, from species choice through final finishing. Advanced characterization techniques are enabling this integration. Advanced non-destructive techniques such as X-ray micro-computed tomography (µCT) are becoming increasingly valuable for identifying species and internal structure of heritage instruments without sampling, exemplifying how modern analysis can inform material choice and understanding prior to treatment [38].

**Figure 4:**
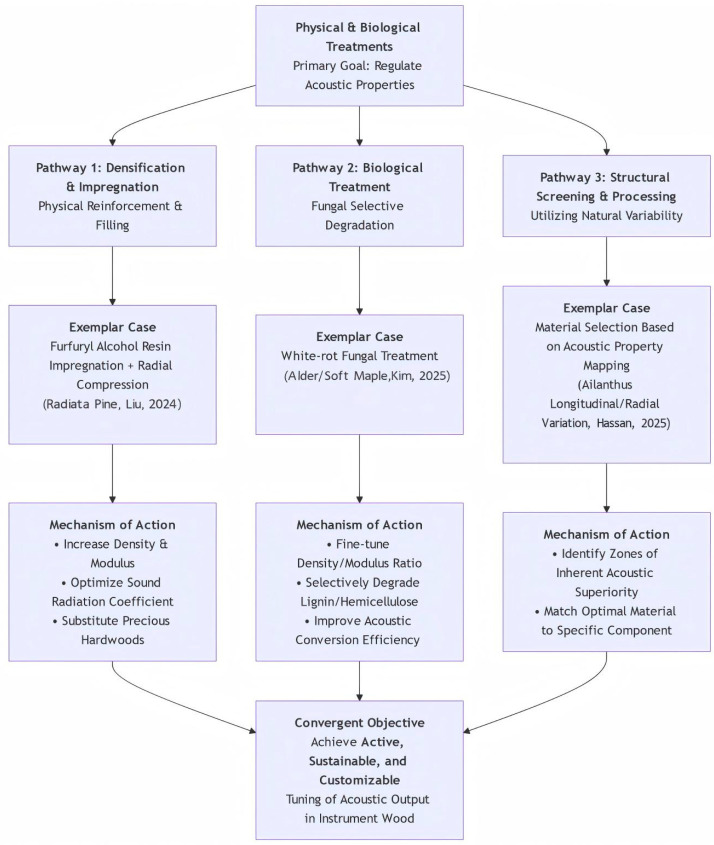
Authors’ own elaboration based on the reviewed literature. Schematic representation of non-conventional physical and biological treatment routes for the acoustic regulation of instrument wood. The diagram contrasts three distinct strategies: (1) densification and impregnation for property enhancement (Liu et al. [17]), (2) biological treatment with white-rot fungi for selective cell wall modification (Kim et al. [32]), and (3) structural screening and processing based on inherent wood variation (Hassan et al. [11]). Although these pathways operate through different mechanisms, they collectively represent a shift beyond passive protection toward active, durable, and potentially tunable acoustic modification.

The diverse methods discussed above are not merely isolated techniques but represent a conceptual evolution. The diverse methods discussed above—ranging from large-scale compression engineering to microscale fungal customization—represent more than a catalog of individual techniques. They signify a conceptual evolution in acoustic modification, wherein wood is no longer merely protected but actively engineered to achieve specific vibrational objectives. This paradigm is synthesized in Figure 4, which illustrates how these varied approaches converge under the unifying concept of intentional material design. From the diagram, it is evident that, whether through bulk property enhancement (such as furfuryl alcohol impregnation and compression [17]), delicate cell wall modification (white-rot fungal treatment [32]), or exploitation of natural diversity (mapping material variations [11]), the common aim is to transcend the limitations of raw material selection. This places instrument makers at the center of the process—not merely as selectors of optimal natural wood, but as potential directors of material behavior. Future developments may involve the hybridization of these approaches. These pathways hold potential for future integration, suggesting hybrid approaches, wherein wood might undergo biological modification before targeted densification in specific regions based on detailed structural maps. Thus, the significance of these alternative treatments lies in collectively expanding the solution space for sustainable instrument making, shifting from dependence on rare, inherently perfect materials toward the intentional creation of optimized materials.

The treatments discussed thus far—chemical, thermal, coating-based, and physical/biological—have each been developed with specific primary functions, be it dimensional stability, surface protection, or acoustic modification. An emerging and more ambitious trend seeks to transcend single-function optimization by integrating multiple capabilities within unified treatment systems, as explored in the following section.

## 5. Multifunctional Integration: Synergistic Strategies for Flame Retardancy, Hydrophobicity, and Sustainability

The preceding sections have examined technologies optimized for individual objectives: chemical modification for dimensional stability, coatings for surface protection, and physical/biological treatments for acoustic tuning. However, real-world instrument applications increasingly demand the simultaneous satisfaction of multiple requirements. Future surface protection technologies for instrument wood will increasingly trend toward multifunctionality, environmental compatibility, and intelligent responsiveness. This represents a departure from single-objective optimization toward more complex, balanced integration of acoustic quality, longevity, aesthetics, and environmental impact.

### 5.1. Flame-Retardant and Preservative Functions with Low-Leaching Integration

Safety requirements for instruments in public venues or transit create additional functional demands. For instruments displayed in public venues or requiring long-distance transportation, flame retardancy and preservation present additional safety considerations. However, conventional flame retardants and preservatives are prone to leaching, raising environmental concerns. Recent research has focused on “anchoring” functional groups to wood through chemical bonding to create low-leach systems. Lin et al. [39,40] developed a treatment system using ammonium dihydrogen phosphate (ADP) and urea: under vacuum-pressure impregnation and heating conditions, phosphate and carbamate groups are grafted onto wood hydroxyl groups, forming covalent bonds (C-O-P). The modified wood maintains an exceptionally high limiting oxygen index (LOI > 80%), with substantially reduced heat release rate even after rigorous water leaching tests, demonstrating effective, low-leach flame-retardant protection. Similarly, treatment with phosphorylated and carbamylated industrial lignin yields wood exhibiting both fire resistance and biological decay resistance [40]. These approaches provide technical prototypes for durable, environmentally benign functional wood modification. Creating stable, non-leachable protective networks is also being explored through other routes, such as silica-lignin hybrid coatings for enhanced durability and moisture resistance [23]. The critical challenge lies in incorporating these multifunctional, immobile treatments without compromising vibrational properties—an aspect that remains poorly understood.

### 5.2. Superhydrophobic and Self-Cleaning Surfaces

**Figure 5:**
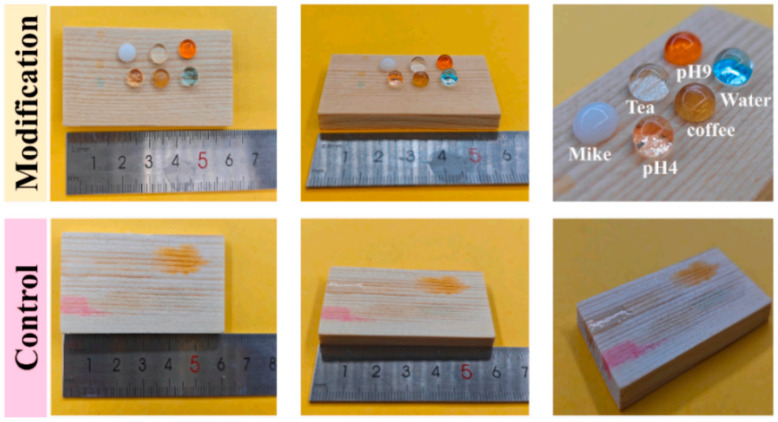
Anti-permeability test diagram: Water droplets exhibit a quasi-spherical morphology on the treated wood surface, whereas they rapidly penetrate the control wood [41].

Inspired by biological systems, superhydrophobic surfaces offer another route to multifunctional protection. Inspired by the “lotus effect,” superhydrophobic surface construction offers one approach to protecting wood against liquid water damage and reducing surface soiling. Xing et al. [41] fabricated a superhydrophobic surface with a water contact angle of 161.2° by spraying a nanocomposite coating comprising palm wax and polydimethylsiloxane (PDMS) onto wood (Figure 5). This coating exhibited good anti-fouling and self-cleaning properties and maintained its superhydrophobic state after abrasion with sandpaper, tape peeling, acid/base immersion, and accelerated UV aging. Yao et al. [42] employed a three-step “spray-sand-spray” process to incorporate silane-modified iron oxide pigments into PDMS/epoxy resin, creating colorful, highly durable superhydrophobic coatings. Such coatings prevent water ingress, reducing dimensional changes associated with wetting–drying cycles. However, the acoustic implications of such surface architectures remain unexplored. However, these studies do not evaluate whether the micro–nano roughness characteristic of superhydrophobic coatings increases sound scattering. Simpler hydrophobic treatments, such as silane-siloxane impregnation, can substantially improve dimensional stability with minimal visual alteration, potentially offering a less intrusive solution [43]. The choice between superhydrophobic “lotus leaf” surfaces and deep-penetrating hydrophobic impregnation depends on the specific balance required among water protection, aesthetic preservation, and acoustic considerations.

### 5.3. Fully Bio-Based and Binder-Free Coatings

At the frontier of sustainability lie coatings derived entirely from renewable resources. Pursuing sustainability to its fullest extent entails developing coatings that require no fossil-derived raw materials or synthetic binders. Kobayashi et al. [30] reported an innovative approach: plant cell wall components—cellulose, hemicellulose, and lignin—were gently disassembled in formic acid and subsequently reassembled into biomass films, which were then hot-pressed onto wood surfaces at temperatures exceeding 200 °C. Hot pressing induces partial pyrolysis of film components and penetration into wood pores, forming a strong, hydrophobic (contact angle >90°) coating without any synthetic polymers or binders. This “all-wood” coating concept—derived from wood and applied to wood—represents an ideal model for circular bioeconomy principles. Chang et al. [29] investigated the use of Acacia confusa heartwood extract as a natural dye and photostabilizer. Treatment with metal mordants such as copper and iron can impart rich coloration while substantially improving light resistance. This work integrates wood protection with natural pigment application and valorization of agricultural and forestry by-products, expanding the scope of sustainable surface treatments. Similarly, using natural extracts such as teak for UV-protective coatings demonstrates how plants’ inherent defense mechanisms against UV radiation can be harnessed for wood protection [44]. These approaches align with the broader framework of “green chemistry” for wood preservation, seeking sustainable solutions within nature’s own repertoire [45].

The strategies summarized in Table 2 represent more than incremental improvements; they signify a transition from sequential to simultaneous multifunctional integration. The design premise shifts to: “How can multiple functions be incorporated within a single, unified system from the outset?” This is evident in the molecular logic of in situ phosphorylation, which intrinsically links flame retardancy and low leaching, and in biomimetic coatings where durability emerges from microstructural design. However, the final column in Table 2 reveals an important tension: the approaches that achieve the strongest integration—forming covalent networks, constructing nano-rough surfaces, and welding biomass films—also introduce the most substantial modifications to a wood’s interfacial mechanics. A paradox emerges: as multifunctional integration becomes more successful, the potential for unintended acoustic consequences increases.

The multifunctional strategies surveyed in this section reveal both significant progress and persistent challenges. While technological capabilities for integration continue to advance, the fundamental tension between protective efficacy and acoustic transparency remains unresolved. These observations naturally lead to consideration of the future directions necessary to address these challenges.

## 6. Future Perspectives and Research Priorities

The preceding chapters have systematically examined the landscape of protective and modification strategies for instrument wood, revealing a field characterized by sophisticated technologies yet constrained by persistent gaps in knowledge and methodology. Synthesizing these findings, several critical priorities emerge for advancing the field from empirical trial-and-error toward predictive, knowledge-based design.

(1) Systematic, cross-scale, multi-objective research.

The first priority addresses the scale mismatch identified throughout this review. There is an urgent need to establish integrated experimental and simulation platforms that span from molecular and cellular levels to complete instruments, including geometrically complex components such as arched soundboards. Future research designs must simultaneously evaluate acoustic performance (damping, vibrational modes, radiation efficiency), durability (water resistance, UV stability, biological resistance), and long-term stability under both accelerated and natural aging conditions. The development of quantitative multi-objective trade-off analysis models is essential for navigating the inherent conflicts between protective efficacy and acoustic optimization. Experimental modal analysis and finite element simulation techniques, as demonstrated in studies on instruments such as the Oud [46], provide promising methodological templates for component-level vibroacoustic validation.

(2) Open-access “Wood–Treatment–Performance” databases.

The second priority responds to the fragmentation of research data across the field. Inspired by materials informatics, the creation of open-access databases that systematically collect, organize, and disseminate performance data (including long-term aging information) for diverse wood species subjected to various treatments would significantly accelerate progress. Such databases could serve as training sets for machine learning models [47,48] to predict outcomes for novel material combinations, optimize treatment parameters, and accelerate the discovery of high-performance solutions. Crucially, these databases should also incorporate information on material sustainability and provenance to align with growing environmental imperatives.

(3) Smart and intelligent surface treatment systems.

The third priority looks toward next-generation dynamic material systems. Future coatings and modifications are likely to be dynamic rather than static. Research into smart material systems capable of adapting their permeability in response to humidity fluctuations, selectively damping specific frequency ranges, or autonomously repairing minor damage [28] may offer transformative approaches to the fundamental tension between protection and acoustic transparency. Bioinspired designs, such as foam-like compressible wood for impact protection [49], illustrate the potential for multifunctional dynamic materials that could reshape the possibilities for instrument wood engineering.

## 7. Conclusions

This critical review has examined the current state of research on protective and modification strategies for instrument wood, synthesizing findings from studies on chemical and thermal modification, coating technologies, physical densification, and biological treatments. Three main conclusions emerge from this analysis.

First, treatment outcomes are highly context-dependent, varying significantly with wood species, treatment parameters, and the specific functional requirements of different instrument components. Second, inherent trade-offs exist between protective efficacy and acoustic performance: improvements in dimensional stability, hydrophobicity, or flame retardancy often come at the expense of vibrational properties, and no single technology universally optimizes all performance dimensions. Third, a persistent gap remains between laboratory-scale research and real-world instrument validation, particularly regarding long-term performance under natural aging conditions.

By systematically identifying these patterns and limitations, this review provides a foundation for more targeted interdisciplinary research. The field stands at a critical juncture: moving forward requires not merely developing new materials but establishing predictive models, intelligent design strategies, and comprehensive assessment standards that will enable truly optimized, sustainable solutions for preserving the world’s resonant woods.

## Figures and Tables

**Table 1:** Novel bio-based and nanocomposite coating yechnologies.

Coating Type	Core Materials	Key Functions	Sustainability	Acoustic Impact	Ref.
Fully bio-based waterproof	Bark extracts (suberinic acids, polyphenols)	Excellent water resistance	100% bio-based, closed-loop	Essentially unstudied	[22]
Lignin-nano ZnO composite	Nanosized lignin + ZnO + PHBV	Superhydrophobic, UV shielding, antifungal	Uses lignin by-product; biodegradable	Lacking systematic study	[23]
CNF reinforced	CNF + PVA matrix	Improves hardness, wear resistance	CNF renewable; water-based	Preliminary exploration	[25]
Natural extract coating	Acacia heartwood extract + mordants	Coloring + photostabilization	Natural pigments, high-value use	Not addressed	[29]
Binder-free biomass film	Reassembled cellulose/hemicellulose/lignin	Strong, hydrophobic coating	Fully plant-derived, no synthetics	Completely blank	[30]
Nano-SiO_2_ enhanced	Modified nano-SiO_2_ + waterborne resin	Enhances hardness, abrasion resistance	Water-based, low VOC	Overlooked	[9]
CNC additive	CNC + waterborne acrylic	Improves decay resistance	Renewable CNC; eco-friendly	Paradigm correct but not deepened	[26]

**Table 2:** Strategies for multifunctional and sustainable surface integration.

Integration Strategy	Exemplary Technology/System	Primary Function Achieved	Additional Functions/Benefits	Sustainability & Environmental Features	Key Challenge/ Research Gap
Flame- retardant & preservative	In situ phosphorylation/carbamylation (adp/urea) [39,40]; hosphorylated lignin [40]	High flame retardancy with lOI above 80% after leaching	Low-leach, durable biological decay resistance	Uses industrial lignin by-product; reduces environmental leaching risk	Acoustic impact largely unknown. Effect of fixed, rigid networks on damping and vibration
Superhydrophobic & self-cleaning	Palm wax/pdms nanocomposite coating [41]; “spray-sand-respray” Colored coating [42]	Water repellency with contact angle above 160°, self-cleaning	Excellent durability against abrasion, chemicals, and UV aging	Potential use of bio-based waxes (e.g., palm wax)	Potential acoustic penalty. Micro-nano roughness may increase sound scattering losses; requires evaluation
Deep penetration hydrophobization	Silane-siloxane impregnation [43]	Improved dimensional stability, bulk hydrophobization	Minimal visual alteration, preserves wood aesthetics	Low VOC options available; durable protection	Acoustic effect presumed minimal but unverified. Needs confirmation of uniform modification
Fully bio-based & binder-free	Reassembled biomass film hot-pressed coating [30]	Strong, hydrophobic coating without synthetics	“All-wood” Concept, pure biomass circularity	100% bio-based, binder-free, ideal circular bioeconomy model	Acoustic role completely unexplored. Unique coating-wood fusion interface’s vibro-acoustic behavior is unstudied.
Natural coloring & stabilization	Acacia confusa heartwood extract + mordants [29]; teak extract [44]	Natural coloration with improved lightfastness	Natural photostabilizers, high-value use of extracts	Renewable plant extracts	Direct acoustic impact likely minor. Part of broader “green chemistry” Toolkit [45] for holistic sustainable design

## Data Availability

No new data were created or analyzed in this study. Data sharing is not applicable.
